# Endotypes of difficult-to-control asthma in inner-city African American children

**DOI:** 10.1371/journal.pone.0180778

**Published:** 2017-07-07

**Authors:** K. R. Brown, R. Z. Krouse, A. Calatroni, C. M. Visness, U. Sivaprasad, C. M. Kercsmar, E. C. Matsui, J. B. West, M. M. Makhija, M. A. Gill, H. Kim, M. Kattan, D. Pillai, J. E. Gern, W. W. Busse, A. Togias, A. H. Liu, G. K. Khurana Hershey

**Affiliations:** 1 Department of Pediatrics, Division of Allergy and Immunology, Cincinnati Children’s Hospital Medical Center, University of Cincinnati, Cincinnati, Ohio, United States of America; 2 Rho Federal Systems Division Inc., Chapel Hill, North Carolina, United States of America; 3 Department of Pediatrics, Division of Asthma Research, Cincinnati Children’s Hospital Medical Center, University of Cincinnati, Cincinnati, Ohio, United States of America; 4 Department of Pediatrics, Division of Pulmonary Medicine, Cincinnati Children’s Hospital Medical Center, University of Cincinnati, Cincinnati, Ohio, United States of America; 5 Department of Pediatrics, Johns Hopkins University School of Medicine, Baltimore, Maryland, United States of America; 6 Boston University School of Medicine, Boston, Massachusetts, United States of America; 7 Ann and Robert H. Lurie Children's Hospital of Chicago, Chicago, Illinois, United States of America; 8 University of Texas Southwestern Medical Center, Dallas, Texas, United States of America; 9 Henry Ford Health System, Detroit, Michigan, United States of America; 10 College of Physicians and Surgeons, Columbia University, New York, New York, United States of America; 11 Children’s National Health System, Washington, District of Columbia, United States of America; 12 University of Wisconsin School of Medicine and Public Health, Madison, Wisconsin, United States of America; 13 National Institute of Allergy and Infectious Diseases, Bethesda, Maryland, United States of America; 14 National Jewish Health, Denver, Colorado, United States of America; 15 Children’s Hospital Colorado and University of Colorado School of Medicine, Aurora, Colorado, United States of America; Forschungszentrum Borstel Leibniz-Zentrum fur Medizin und Biowissenschaften, GERMANY

## Abstract

African Americans have higher rates of asthma prevalence, morbidity, and mortality in comparison with other racial groups. We sought to characterize endotypes of childhood asthma severity in African American patients in an inner-city pediatric asthma population. Baseline blood neutrophils, blood eosinophils, and 38 serum cytokine levels were measured in a sample of 235 asthmatic children (6–17 years) enrolled in the NIAID (National Institute of Allergy and Infectious Diseases)-sponsored Asthma Phenotypes in the Inner City (APIC) study (ICAC (Inner City Asthma Consortium)-19). Cytokines were quantified using a MILLIPLEX panel and analyzed on a Luminex analyzer. Patients were classified as Easy-to-Control or Difficult-to-Control based on the required dose of controller medications over one year of prospective management. A multivariate variable selection procedure was used to select cytokines associated with Difficult-to-Control versus Easy-to-Control asthma, adjusting for age, sex, blood eosinophils, and blood neutrophils. In inner-city African American children, 12 cytokines were significant predictors of Difficult-to-Control asthma (n = 235). CXCL-1, IL-5, IL-8, and IL-17A were positively associated with Difficult-to-Control asthma, while IL-4 and IL-13 were positively associated with Easy-to-Control asthma. Using likelihood ratio testing, it was observed that in addition to blood eosinophils and neutrophils, serum cytokines improved the fit of the model. In an inner-city pediatric population, serum cytokines significantly contributed to the definition of Difficult-to-Control asthma endotypes in African American children. Mixed responses characterized by T_H_2 (IL-5) and T_H_17-associated cytokines were associated with Difficult-to-Control asthma. Collectively, these data may contribute to risk stratification of Difficult-to-Control asthma in the African American population.

## Introduction

Asthma is a heterogeneous disease comprised of varying clinical, cellular, and molecular phenotypes[1]. Cluster analyses of well-characterized asthma cohorts have identified several distinct clinical phenotypes in adults and children, but they have limited ability to improve management or guide treatment choices because the phenotypes often overlap and do not necessarily relate to specific underlying pathophysiologic mechanisms[2–4]. Further, although cluster studies have identified distinct molecular phenotypes of severe asthma, these phenotypes have not proven to vary by race despite the differing clinical phenotypes. Recently, it has been suggested that the asthma burden seen in urban US populations may be largely explained by demographic factors such as race rather than by environmental factors secondary to living in an urban location[5, 6]. Accordingly, a comparable prevalence of asthma was found in urban and rural populations of African Americans teenagers[7]. African Americans have higher rates of asthma prevalence, morbidity, and mortality in comparison with other racial groups in America[8–10]. It has been suggested that this racial disparity cannot be explained solely by socioeconomic or environmental factors with black race being reported as an independent risk factor for asthma[11, 12]. Collectively, these findings suggest that immunologic profiles that characterize asthma in African Americans may predispose to more severe or difficult to control phenotypes. Interestingly, higher T_H_17 activation was reported in Asians versus European Americans with atopic dermatitis, suggesting a racial difference in immunologic mechanisms of disease[13].

The Asthma Phenotypes in Children (APIC) Study, which was conducted by the NIAID-funded Inner City Asthma Consortium, offered us an opportunity to specifically characterize asthma immunophenotypes in African American children as 64% of APIC participants are African American. Towards this goal, we characterized serum cytokine profiles in a subset of 235 children out of the 717 children (33%) enrolled in APIC. Our findings indicate that serum cytokine concentrations, in addition to blood neutrophils and eosinophils, may be helpful in defining Difficult-to-Control asthma endotypes in African American inner-city children.

## Methods

### Study population

IRB approval was obtained from the following institutions: Johns Hopkins University School of Medicine, Department of Pediatrics, Baltimore, MD; Boston University School of Medicine, Boston, MA; Ann and Robert H. Lurie Children's Hospital of Chicago, Chicago, IL; Cincinnati Children's Hospital Medical Center, Department of Pediatrics, University of Cincinnati, Cincinnati, OH; University of Texas Southwestern Medical Center, Dallas, TX; National Jewish Health, Denver, CO, and Children's Hospital Colorado and University of Colorado School of Medicine, Aurora, CO; Henry Ford Health System, Detroit, MI; College of Physicians and Surgeons, Columbia University, New York, NY; and Children's National Health System, Washington, DC. Written informed consent was obtained from the parent or legal guardian of each child. The subjects included in this study were enrolled in the APIC Study which is an epidemiologic, multi-center, longitudinal study designed to define phenotypic characteristics of Difficult-to-Control asthma among children receiving one year of guideline-based therapy for asthma and rhinitis/rhinosinusitis. A detailed description of study methods including recruitment, enrollment, and data collection (skin testing, spirometry, and exhaled nitric oxide) is described in the primary manuscript for the APIC study[14]. Seven hundred and seventeen subjects from 9 clinical sites (Baltimore MD, Boston MA, Chicago IL, Cincinnati OH, Dallas TX, Denver CO, Detroit MI, New York NY, Washington DC) were enrolled in APIC. Study approval was granted by each center’s Institutional Review Board. Enrolled children were between the ages of 6 to 17 years with physician-diagnosed asthma. Participants were required to report 2 or more episodes of short-acting beta-agonist administration within the 12 months prior to the screening visit (exclusive of exercise-induced symptom use), have insurance coverage, and reside in a pre-selected urban recruitment census to qualify for entry into the study. Only participants who identified as non-Hispanic African American were included in this analysis and 235 subjects were enrolled as suggested by sample size calculations necessary for statistical power of at least 90% based on our previously published results[15]. Comparative data for other races were not included because, of the remaining 129 subjects, only 6 subjects were non-Hispanic white, 17 were of other or mixed race, and 106 were Hispanic with race unidentified.

Protocol-defined asthma and rhinitis management was started at the time of screening. Asthma and rhinitis treatment algorithms were based on the NAEPP Expert Panel Report-3 (EPR-3) and the Rhinitis and its Impact on Asthma (ARIA) 2008 guidelines[16, 17]. Based on study treatment guidelines for asthma and rhinitis, patients were provided with prescribed medications (Flovent® Diskus® (fluticasone), Advair™ Diskus (fluticasone propionate, salmeterol), Ventolin® HFA (albuterol), Flonase® Nasal Spray (fluticasone), cetirizine, montelukast). Demonstration of medication adherence was required for study enrollment and was assessed at the enrollment visit following a 4-week run-in period. Subsequently, asthma control was evaluated at 6 clinical study visits at 2-month intervals at which time clinicians prescribed medications based on the level of asthma control and medication compliance over the previous two months. Asthma control was assessed based on asthma symptoms, albuterol use, use of systemic corticosteroids, FEV_1_ (forced expiratory volume), and current asthma therapy. Classification of subjects occurred following the last study visit. Participants requiring ≤ 50 mcg twice daily of fluticasone, montelukast only, or needing no controller medication at four or more of the six post-baseline study visits, were classified as having Easy-to-Control asthma. Children requiring ≥ 250 mcg twice daily of fluticasone at four or more of six post-baseline study visits were classified as Difficult-to-Control asthma. Children who did not fall into either classification were considered indeterminate and were not included in the analysis.

Of the 717 subjects enrolled in APIC, 510 randomly selected subjects had blood analyzed for cytokines. Of these 510 subjects, 502 had complete data for demographics, blood eosinophils, blood neutrophils, and serum cytokines. Less than 4 baseline visits were done in 38 patients and an additional 100 patients were excluded from our analysis as their treatment classification was indeterminate. Out of these 364 subjects, 235 were African American, of which 108 subjects were classified as Easy-to-Control and 127 subjects were classified as Difficult-to-Control.

### Blood collection and analysis

Whole blood was collected by venipuncture at the enrollment visit. Blood for cytokine analysis was collected into serum separator tubes and centrifuged and aliquoted into cryovials following a standard protocol during or immediately following the study visit. Serum aliquots were frozen and stored at -80°C pending shipment to the biorepository and subsequently to the analyzing laboratory. As freeze-thaw cycles can alter cytokine measures, samples in our analysis were not freeze-thawed[18]. Total numbers of eosinophils and neutrophils were determined by the product of the differential and total cell count. Using 25 μL of participant serum, cytokines were quantified according to the manufacturer’s instructions using a premixed 38 Plex MILLIPLEX MAP Human Cytokine/Chemokine Magnetic Bead Panel (Millipore Corporation, Billerica, MA) and analyzed on a Luminex 100/200 analyzer (BioRad, Hercules, CA). Serum cytokine concentrations were determined using Bio-plex 6.1 software (BioRad, Hercules, CA). A similar approach for analyzing cytokines in BAL (bronchoalveolar lavage) fluid from asthmatics has been used previously[19]. Cytokines included in the analysis are listed in S1 Table.

### Statistical analysis

As shown in S1 Fig, cytokine replicate pairs had a good agreement and were averaged to produce a single result for analysis. Cytokine values were skewed based on a diagnostic plot (S2 Fig) and the result of a Shapiro-Wilk test and were, therefore, log-transformed for statistical tests and models. For analysis purposes, results below detection were assigned a value of the lower limit of detection divided by the square root of 2, and results above detection were assigned a value equal to the upper limit of detection[20].Overall, 22% of cytokine values were below the lower limit of detection and 2% were above the upper limit of detection. The percentage of values above the lower limit of detection by inflammatory marker is displayed in S1 Table and S2 Fig.

To compare differences in baseline characteristics and demographics between Difficult-to-Control and Easy-to-Control asthma, chi-square or Fisher exact tests were used for categorical variables, and t-tests or Wilcoxon tests were used for normally distributed and non-normally distributed continuous variables, respectively. Normality of baseline characteristics and demographics was determined by assessing the skewness of variables. Univariate logistic regression was used to estimate the association with Difficult-to-Control asthma for blood eosinophils, neutrophils, and each of the 38 cytokines. Due to the exploratory nature of the analysis, no attempts were made to adjust for multiple comparisons.

Logistic models were fit using Difficult-to-Control versus Easy-to-Control as the outcome and demographics, blood eosinophils, blood neutrophils, and/or cytokines as covariates. Nested models were compared using likelihood ratio testing, which compares the goodness of fit of two models. The glmnet procedure was used to fit a multiple logistic regression model with an elastic-net penalty, which is a combination of lasso and ridge regression penalties. This technique is particularly efficient for feature selection in data with collinearity and/or many features, in order to select the most relevant cytokines for distinguishing Easy-to-Control versus Difficult-to-Control asthma after adjusting for age, sex, blood eosinophils, and blood neutrophils[21, 22]. Adjustments were performed by including the listed variables in the model, so that the relationships between cytokines and Difficult-to-control asthma were free of confounding. Repeated 10-fold cross validation was used to prevent overfitting and select the best penalty parameters. R version 3.3.3 was used for analyses.

## Results

### Demographic and baseline characteristics of study population

The African American children included in this study were a randomly selected subset of subjects in the APIC study conducted by the Inner City Asthma Consortium (ICAC)[14]. Demographic data from the 235 children comprising our study subset were compared to the entire African American APIC population (N = 311) and no significant differences were observed (data included in S2 Table). Demographic features and baseline characteristics were compared between the Difficult-to-Control and Easy-to-Control populations in our analysis population (Table 1). When compared to Easy-to-Control, Difficult-to-Control participants reported use of more asthma controller medications (p<0.001), had higher rates of oral corticosteroid courses for asthma (p<0.0001), higher total serum IgE (p = 0.004), and lower FEV_1_ (p<0.001) and FEV_1_/FVC (p<0.001) at study entry. Difficult-to-Control participants were more likely to have allergic rhinitis (p<0.001) as well as aeroallergen (p = 0.005) sensitization at study entry. No differences were observed between the Easy-to-Control and Difficult-to-Control groups with regards to sex, age, income, BMI (body mass index) percentile, family history of asthma, age at asthma diagnosis, personal diagnosis of eczema, asthma-related hospitalizations, FeNO (fractional exhaled nitric oxide), or food sensitization.

**Table 1:** Participant characteristics and demographics^1^^,^^2^.

Characteristic	Difficult-to-Control (N = 127)	Easy-to-Control (N = 108)	P-value
**Sex—Male**	67 (52.8%)	64 (59.3%)	0.39
**Age (years)**	11.1 (3.0)	11.0 (2.9)	0.91
**BMI percentile^3^**	89.0 [64.8; 98.7]	87.9 [56.5; 97.2]	0.13
**Income <$15,000**	63 (50.0%)	56 (51.9%)	0.88
**Family history of asthma**	98 (78.4%)	74 (70.5%)	0.22
**Eczema diagnosis**	83 (65.4%)	63 (58.3%)	0.33
**Allergic rhinitis diagnosis**	102 (80.3%)	63 (58.3%)	<0.001
**Asthma medical history**			
**Age at asthma diagnosis (months) ^3^**	24.0 [10.5; 60.0]	24.0 [12.0; 66.0]	0.09
**Controller treatment step**	4.7 (1.5)	1.8 (1.5)	<0.001
**Any hospitalizations (in previous year)**	26 (20.5%)	9 (8.3%)	0.02
**Any steroid courses^4^ (in previous year)**	83 (65.4%)	33 (30.6%)	<0.001
**FeNO (ppb)^3^^,^^5^**	24.5 [12.0; 40.0]	19.0 [11.9; 34.5]	0.17
**FEV_1_ (% predicted)**	87.3 (17.9)	97.4 (15.9)	<0.001
**FEV_1_/FVC (% predicted)**	73.3 (11.0)	79.5 (8.4)	<0.001
**Total serum IgE (kU/L)^3^^,^^5^**	412.5 [106.8; 957.5]	185.5 [61.0; 576.2]	0.004
**Number of allergen sensitizations^6^**	10.2 (6.2)	8.1 (6.1)	0.008
**Number of aeroallergen sensitizations^7^**	9.5 (5.6)	7.4 (5.6)	0.005
**Sensitized to foods^8^**	35 (27.8%)	36 (33.6%)	0.41

1. Unless otherwise noted, characteristics are compared using chi-square or Fisher’s exact test for categorical variables, and ANOVA or t-test for continuous variables.

2. Unless otherwise noted, summary statistics are frequency (%) for categorical variables and mean and standard deviation for continuous variables. Unless otherwise noted, data was obtained at screening visit.

3. Summarized using the median and inter-quartile range and tested using a Wilcoxon test.

4. Defined as having at least one outpatient oral steroid course or hospitalization.

5. Obtained at the time of enrollment visit.

6. Sensitization is based on a positive skin prick test and/or positive specific IgE (≥0.35 kUA/L) to at least one of the following allergens: *Alternaria tenuis* (skin prick test) or *Alternaria alternata* (specific IgE), *Aspergillus fumigatus* (both skin prick test and specific IgE), *Cladosporium herbarum* (specific IgE only), *Dermatophagoides farinae*, *Dermatophagoides pteronyssinus*, German cockroach, American cockroach, mouse, rat, cat, dog, oak, pecan, birch, maple, Eastern 8 tree mix, ragweed mix (giant/short; skin prick test) or short ragweed (specific IgE), timothy grass, Kentucky Blue/June, Orchard and Timothy (K-O-T) grass mix, peanut, egg and milk.

7. Sensitization is based on a positive skin prick test and/or positive specific IgE (≥0.35 kUA/L) to at least one of the following allergens: *Cladosporium herbarum* (specific IgE only), *Dermatophagoides farinae*, *Dermatophagoides pteronyssinus*, German cockroach, American cockroach, mouse, rat, cat, dog, oak, pecan, birch, maple, Eastern 8 tree mix, ragweed mix (giant/short; skin prick test) or short ragweed (specific IgE), timothy grass, Kentucky Blue/June, and Orchard and Timothy (K-O-T) grass mix.

8. Sensitization is based on a positive Fx5 multitest which includes specific IgE testing to the following allergens: wheat, egg, milk, soy, peanut, and fish.

### Blood concentrations of eosinophils and neutrophils differ by treatment classification

**Fig 1:**
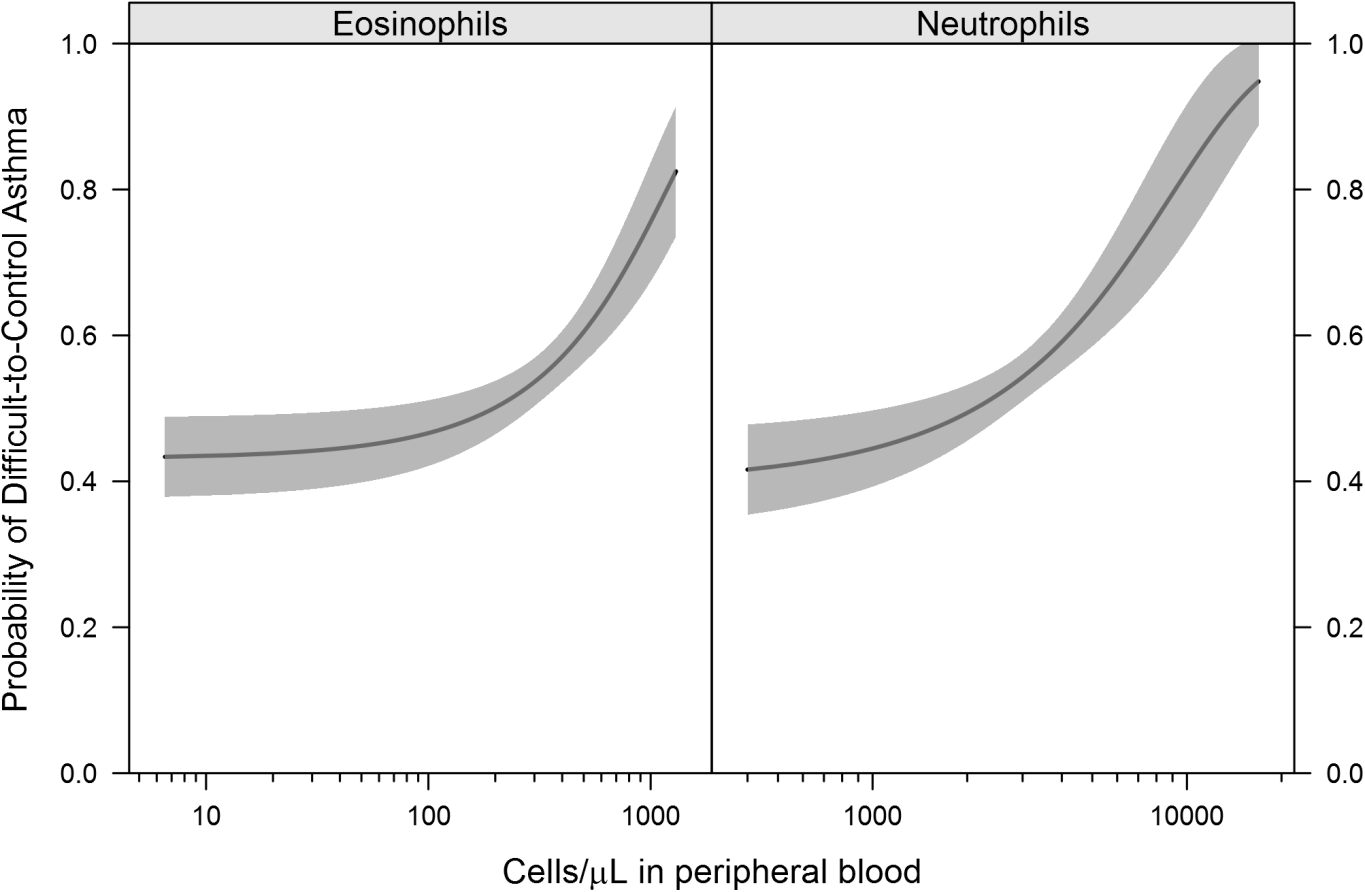
Probability of difficult-to-control asthma rises as blood eosinophil and neutrophil concentrations increase. Graphical representation of blood eosinophil and neutrophil concentrations and the probability of Difficult-to-Control asthma. Values from all 235 subjects in the analysis are included. The blood neutrophil and eosinophil counts were not correlated in this population (Pearson correlation = 0.11, p = 0.11).

Children with Difficult-to-Control asthma had higher numbers of blood eosinophils (p = 0.02) and neutrophils (p = 0.02) (Table 2) when compared to children with Easy-to-Control asthma. There was no correlation between eosinophil and neutrophil counts. The probability of Difficult-to-Control asthma was greater as blood eosinophil and neutrophil concentrations increased (Fig 1). Since systemic corticosteroids can alter blood neutrophil and eosinophil counts, we examined corticosteroid use among participants. Seven percent of subjects received an oral corticosteroid course in the month prior to their blood draw. Receiving an oral corticosteroid course in the month prior to the blood draw was not associated with significantly elevated or lower neutrophil

**Table 2:** Concentrations of blood eosinophils and neutrophils by treatment classification^1^.

Granulocyte	Difficult-to-Control (n = 127)	Easy-to-Control (n = 108)	OR [95% CI]^2^	P-value^3^
**Eosinophils/μL**	351 (252)	278 (210)	1.43 (1.07, 1.95)	0.02
**Neutrophils/μL**	3248 (1998)	2712 (1437)	1.40 (1.06, 1.91)	0.02

1. Numbers shown for Difficult-to-Control and Easy-to-Control are Mean (SD).

2. OR = odds ratio associated with a 1-interquartile range change; CI = confidence interval.

3. P-value based on t-test.

### Serum cytokine concentrations differ by treatment classification

IL-12p40 (p = 0.005), IL-13 (p = 0.04), IL-1β (p = 0.02), and IL-4 (p = 0.03) were increased in those with Easy-to-Control asthma (Table 3). No cytokines were positively associated with Difficult-to-Control asthma. A multivariate feature selection procedure was used to determine which cytokines were most predictive of Difficult-to-Control versus Easy-to-Control asthma (Table 4). IL-5, IFN-γ (interferon gamma) and T_H_17 (CXCL-1, IL-17A, IL-8) associated cytokines were positively associated with Difficult-to-Control asthma; while EGF (epidermal growth factor), eotaxin, IL-lβ, IL-4, IL-12p40, IL-13, and MDC (macrophage-derived chemokine) were positively associated with Easy-to-Control asthma.

**Table 3:** Serum concentrations of 38 cytokines by treatment classification^1^.

Cytokine (pg/mL)	Difficult-to-Control (n = 127)	Easy-to-Control (n = 108)	OR [95% CI]^2^	P-value^3^
CXCL-1	2129.8 (1.5)	1931.0 (1.6)	2.99 (0.81, 11.4)	0.10
EGF	92.8 (3.2)	108.3 (3.0)	0.76 (0.45, 1.28)	0.30
EOTAXIN	61.9 (1.8)	68.5 (1.7)	0.46 (0.15, 1.36)	0.16
FGF2	58.0 (1.8)	61.6 (2.0)	0.71 (0.28, 1.79)	0.47
FLT3L	9.5 (3.8)	10.4 (3.5)	0.88 (0.56, 1.40)	0.60
FRACTALKINE	101.3 (3.0)	96.3 (2.3)	1.13 (0.62, 2.08)	0.70
GCSF	40.7 (2.2)	40.4 (2.4)	1.03 (0.50, 2.12)	0.94
GMCSF	25.9 (2.0)	28.7 (2.2)	0.65 (0.29, 1.43)	0.29
IFNALPHA2	43.9 (2.9)	46.2 (2.7)	0.89 (0.50, 1.59)	0.70
IFNGAMMA	14.6 (4.9)	12.8 (4.7)	1.14 (0.78, 1.66)	0.51
IL10	2.6 (3.7)	3.6 (5.2)	0.71 (0.47, 1.07)	0.10
**IL12P40**	**12.6 (3.2)**	**20.1 (3.6)**	**0.49 (0.29, 0.79)**	**0.005**
IL12P70	3.3 (4.4)	3.7 (5.9)	0.90 (0.63, 1.30)	0.59
**IL13**	**4.1 (5.9)**	**6.9 (7.1)**	**0.71 (0.51, 0.97)**	**0.04**
IL15	1.6 (2.7)	2.1 (3.2)	0.61 (0.35, 1.05)	0.08
IL17A	5.8 (6.0)	4.4 (5.9)	1.22 (0.87, 1.71)	0.25
IL1ALPHA	17.9 (3.8)	21.7 (4.1)	0.79 (0.51, 1.22)	0.28
**IL1BETA**	**1.1 (2.8)**	**1.6 (3.7)**	**0.55 (0.32, 0.91)**	**0.02**
IL1RA	69.8 (2.8)	76.1 (2.6)	0.82 (0.45, 1.48)	0.51
IL2	2.0 (3.7)	2.7 (4.2)	0.71 (0.46, 1.10)	0.12
IL3	0.6 (1.5)	0.6 (1.6)	0.33 (0.07, 1.32)	0.13
**IL4**	**5.1 (3.1)**	**7.5 (4.4)**	**0.60 (0.37, 0.95)**	**0.03**
IL5	1.0 (3.4)	0.9 (3.5)	1.33 (0.82, 2.22)	0.25
IL6	2.7 (5.6)	3.6 (7.2)	0.81 (0.59, 1.12)	0.20
IL7	6.4 (3.0)	6.5 (3.4)	0.98 (0.58, 1.64)	0.93
IL8	22.1 (3.4)	18.5 (3.2)	1.34 (0.81, 2.24)	0.26
IL9	1.7 (2.8)	1.8 (2.5)	0.98 (0.53, 1.81)	0.94
IP10	301.3 (1.7)	315.8 (1.8)	0.71 (0.25, 2.04)	0.52
MCP1	396.7 (1.7)	390.2 (1.7)	1.16 (0.36, 3.78)	0.81
MCP3	18.4 (2.6)	21.7 (2.5)	0.66 (0.35, 1.23)	0.19
MDC	1893.9 (1.5)	1988.8 (1.4)	0.43 (0.07, 2.16)	0.32
MIP1ALPHA	18.8 (4.0)	18.8 (3.7)	1.00 (0.65, 1.56)	>0.99
MIP1BETA	50.8 (2.0)	51.0 (1.9)	0.98 (0.40, 2.39)	0.96
SCD40L	9321.5 (1.3)	8952.1 (1.5)	2.18 (0.39, 14.0)	0.38
TGFALPHA	2.8 (3.1)	2.4 (3.1)	1.40 (0.83, 2.39)	0.21
TNFALPHA	11.3 (2.0)	11.2 (2.3)	1.02 (0.46, 2.23)	0.96
TNFBETA	3.5 (5.2)	5.3 (5.8)	0.72 (0.51, 1.03)	0.07
VEGF	268.8 (2.3)	279.9 (2.4)	0.87 (0.43, 1.78)	0.71

1. Numbers shown for Difficult-to-Control and Easy-to-Control are Geometric Mean (Geometric SD).

2. OR = odds ratio associated with a 1-log10 unit change; CI = confidence interval.

3. P-value based on t-test.

**Table 4:** Inflammatory mediators associated with asthma control based on multivariate feature selection.

Selected Inflammatory Mediators (direction of association with Difficult-to-Control asthma)^1^		
T_H_17 associated	T_H_2 associated	Non T_H_2 or T_H_17 associated
• CXCL-1 (+) • IL-8 (+) • IL-17A (+)	• IL-4 (-) • IL-5 (+) • IL-13 (-)	• EGF (-) • Eotaxin (-) • IFN-γ (+) • IL-1β (-) • IL-12p40 (-) • MDC (-)

1. + denotes cytokines associated with Difficult-to-Control disease,—denotes cytokines associated with Easy-to-Control asthma.

### Definition of Difficult-to-Control childhood asthma endotypes using blood eosinophils, neutrophils, and serum cytokines

To ascertain whether cytokines bring added value to the definition of the Difficult-to-Control childhood asthma endotype in our African American cohort, models with the outcome of Difficult-to-Control asthma were compared using likelihood ratio tests (Table 5). Age, sex, blood eosinophils, and neutrophils, and/or selected serum cytokines were included in all models. Cytokines selected by the multivariate feature selection procedure were the only cytokines included in the models. The model including only demographics was not significantly different than the null model. The addition of blood eosinophils and neutrophils significantly improved the fit of the model (p = 0.006). The addition of selected cytokines further improved the fit of the model when compared to the model containing only demographics and blood eosinophils and neutrophils (p<0.001). Thus the addition of CXCL-1, IFN-γ, IL-5, IL-17A, EGF, eotaxin, IL-lβ, IL-4, IL-12p40, IL-13, and MDC serum concentrations to blood eosinophils and neutrophils adds significant value to the definition of the Difficult-to-Control asthma endotype.

**Table 5:** In addition to demographics and blood eosinophils and neutrophils, serum cytokines strengthen the characterization of difficult-to-control asthma.^1^^,^^2^.

Model	Variables	Comparison	Area Under the Curve (95% CI)	P-value^3^
**M0**	Null model	—	—	—
**M1**	Demographics^4^	vs. M0	0.53 (0.45, 0.60)	0.61
**M2**	Demographics + eosinophils/neutrophils	vs. M1	0.62 (0.55, 0.69)	0.006
**M3**	Demographics + eosinophils/neutrophils + selected cytokines^5^	vs. M2	0.75 (0.69, 0.81)	<0.001

1. Models including demographics, blood eosinophils, and neutrophils, and/or selected serum cytokines with the outcome of Difficult-to-Control asthma were compared using likelihood ratios.

2. Additional results not shown in table

A) Model with selected cytokines only: AUC = 0.71 (95% CI: 0.64, 0.77)

B) Model with demographics + selected cytokines: AUC = 0.71 (95% CI: 0.64, 0.78).

C) In a test of the model with demographics + selected cytokines versus the model with demographics only (M1 in the table), the resulting p-value was 0.001.

3. P-value based on likelihood ratio test.

4. Demographics include age and sex.

5. Selected cytokines included only cytokines selected by the multivariate feature selection procedure (CXCL-1, IL-8, IFN-γ, IL-5, IL-17A, EGF, eotaxin, IL-lβ, IL-4, IL-12p40, IL-13, and MDC).

## Discussion

Despite the strong racial disparities observed in asthma morbidity, previous studies have not characterized immunophenotypes of Difficult-to-Control asthma in African American children[23, 24]. We found that although blood eosinophils and neutrophils were useful biomarkers of Difficult-to-Control asthma, serum cytokines significantly enhanced the definition of Difficult-to-Control asthma. Mixed responses characterized by IL-5 and IL-17 upregulation were associated with Difficult-to-Control disease while IL-4 and IL-13 were associated with Easy-to-Control asthma. This data may contribute to the ability to risk stratify this population of Difficult-to-Control asthmatics. These findings may have important treatment implications as they suggest consideration of the use of biologics to target multiple inflammatory pathways in African American children with Difficult-to-Control asthma.

Increased eosinophils or neutrophils have been reported in sputum of severe and poorly controlled asthmatics[25, 26] and increased blood neutrophils and eosinophils have been associated with severe asthma in children[27]. Similarly, we found increased blood neutrophils and eosinophils in the Difficult-to-Control population.

Difficult-to-Control asthma was positively associated with cytokines/chemokines (CXCL-1, IL-17A, and IL-8) previously described to be associated with neutrophilic inflammation. Association of increased IL-8 with severe asthma has previously been reported in adults and children[19, 26, 28]. Previous analysis of airway secretion samples from predominantly African American children with asthma showed that, in addition to other cytokines and chemokines, Differential CXCL-1 and IL-8 concentrations characterized severe compared to moderate asthma[19]. Increased IL-17A has been implicated in severe asthma by prior studies[29, 30].

Traditionally, T_H_2-associated inflammation has been implicated in the pathogenesis of asthma [31]. In our study, IL-4 and IL-13 were positively associated with Easy-to-Control asthma. Primarily T_H_2-associated asthma is generally glucocorticoid responsive, while T_H_2/T_H_17 predominant asthma may be resistant to corticosteroid treatment[32, 33]. In our population, Difficult-to-Control asthma was positively associated with a combination of T_H_2 and T_H_17-associated inflammatory markers. T_H_17 inflammation has been associated with neutrophilic inflammation and corticosteroid unresponsive asthma[30, 34]. Recent data from our group and others suggests that this may actually be due to a unique population of cells that co-produce T_H_2 and T_H_17 cytokines[15, 33, 35]. Brandt et al. reported the presence of IL-13+IL-17A+ double producing T-cells in the lungs of mice co-exposed to house dust mite and diesel exhaust particles[35]. Studies have implicated dual-positive T_H_2/T_H_17 cells in steroid resistant asthma. Irvin et al. analyzed the infiltrating T helper cells in the BAL fluid from asthmatic patients and assessed their response to steroid treatment *in vitro*[33]. They found that dual-positive T_H_2/T_H_17 (IL-4+/IL-17+) cells and IL-17A were present at a higher frequency in the BALF from steroid-resistant asthmatic patients. These T_H_2/T_H_17 cells were resistant to DEX (dexamethasone)-induced cell death during an *in vitro* incubation and the T_H_2/T_H_17 predominant subgroup of patients manifested the most severe form of asthma[33]. We found an association between IL-17 and IL-5 with difficult-to-control asthma, in comparison to previous studies which report IL-4+IL-17+ or IL-13IL-17+ T-cell populations. T_H_2 cells are classically regarded as a homogenous population, however, multiple recent studies have challenged this notion. Notably, the only partial overlap between IL-4+ and IL-5+ cells was observed in CD4+163- cells from BAL of asthmatics in the study by Irvin et al. This suggests there is some degree of nonsynchronous production of T_H_2 cytokines in T-cell populations, which may in part explain our T_H_2-asscoiated cytokine findings. Further, pathogenic IL-5+ T_H_2 cells have been shown to be central to the development of eosinophilic airway inflammation and possess a greater ability to promote inflammation[36].

We were unable to compare immunophenotypes between racial groups as the APIC population is 64% African American with the remainder of the population being racially heterogeneous. However, analysis of a smaller and heterogeneous population of non-African American in the APIC study showed a differing cytokine profile, which urges consideration of whether Difficult-to-Control asthma phenotypes differ by race. Further validation of these findings in another cohort and comparison to a non-African American comparison group should be pursued. Previous reports have shown that the effect sizes of genetic variants which relate to asthma significantly differ between non-European and European populations[37]. Notably, racial differences in T_H_2/T_H_17 inflammatory pathways have been reported in atopic dermatitis whereby Asian patients had a higher induction of T_H_17 /T_H_22-associated cytokines compared to patients of European ancestry[13].

In this study, we focused on African Americans, however, data from 129 non-African American APIC subjects were analyzed using the same statistical procedures. The racial heterogeneity and small size of this population in comparison to the African American population limited the reliability of this data, which was not included in the manuscript for this reason. However, it is interesting to note that a different cytokine profile was identified in the non-African American population. IL-1RA was positively associated with Difficult-to-Control disease and IL-5 was positively associated with Easy-to-Control asthma.

Our study has several unique strengths. These include features inherent to the APIC study from which the patients were enrolled. APIC enrolled inner-city children, a population disproportionately affected by asthma morbidity and mortality[38]. Since the majority of APIC study participants were African American, we were positioned to characterize inflammatory profiles in at-risk African American children with asthma. APIC is a multi-center, longitudinal study, which allowed for a prospective evaluation over an entire year, designed to assess numerous parameters to identify factors that promote Difficult-to-Control asthma. APIC subjects received standardized, algorithm-based management of asthma and allergic rhinitis for one year. Further, peripheral blood characterization was obtained at baseline, which allows consideration for validation of this method to be used as a predictive model for assessing asthma control in a clinical setting. Additionally, blood samples are more likely to reflect systemic inflammation than bronchial biopsies or washings and have been previously reported to be associated with airway inflammation, asthma phenotype, and asthma severity[39, 40].

Our study also has some important limitations. We cannot control for the potential effects of inhaled or oral corticosteroids on the concentrations of chemokines, cytokines, and granulocytes observed in our study. Seven percent of subjects in our population were on an oral corticosteroid in the month prior to their blood draw. We did not see any impact of oral corticosteroids on blood neutrophils or eosinophils in this subset and oral corticosteroids are more likely to alter blood granulocytes than inhaled corticosteroids in the month prior to their blood draw. Furthermore, use of blood samples for testing for granulocyte and inflammatory mediators, as in our study, would, in theory, be less susceptible to the effects of inhaled corticosteroids when compared to studies utilizing samples obtained from the airways (sputum, BAL, bronchial tissue). A previous study showed no association between the use of inhaled corticosteroids and sputum neutrophil percentage[24]. Also, Hastie et al. reported no significant effect of inhaled corticosteroid use on sputum cell counts or inflammatory mediator levels[41].

Additionally, as is true with other studies assessing cytokines, cytokine concentrations are relatively low which can lead to difficulty in detection. Percent of values above the lower limit of detection for cytokines measured in the study are included in S1 Table and are comparable to levels seen in other studies. Our analysis did not account for potential confounders which may have impacted specific cytokine values such as season in which blood was drawn and the presence of acute respiratory infection. However, the degree of influence that season has on inflammatory cells and cytokines is unclear with previous research suggesting there was no alteration in T_H_17 and T_H_1 responses during pollen season in patients with allergic respiratory disease[42]. Also, due to the exploratory nature of this analysis, we did not adjust for multiplicity in the univariate tests shown in Table 3. Although we acknowledge that the resulting p-values insufficiently control for type I error (false positives), they have minimal contribution to the main analysis, which is the prediction model. We feel that the methodology used for building the prediction model adequately addresses the issue of multiplicity. Finally, as we measured serum cytokine concentrations and did not isolate and phenotype T-cells, we cannot definitively prove that the cytokines were secreted from T_H_-cells as opposed to other immune cells.

The need for risk stratification, refinement of the definition of asthma endotypes, and novel therapeutic agents that target the biological mechanisms underlying the severe asthma phenotype has been highlighted by experts[43]. Results of this study can be used in conjunction with other research to improve risk stratification in this asthmatic population. The data herein urge the consideration of using multiple biologic treatments for Difficult-to-Control asthma in African American children. Given the association of IL-17A and IL-5 with Difficult-to-Control asthma in African American children in our inner-city population, therapeutics targeting IL-17 and IL-5 should be considered. A trial of brodalumab, a human anti–IL-17 receptor A monoclonal antibody, in poorly controlled moderate to severe asthmatics did not show significant improvement in primary or secondary endpoints[44]. However, it may show efficacy in a trial of African American asthmatics with elevated serum IL-17. Further, our data suggest that anti-IL-5 may be most effective in African Americans. Targeting both T_H_2 and T_H_17 associated pathways of inflammation has been suggested previously given the concern that isolated targeting of T_H_2 cytokines may promote T_H_17-associated corticosteroid resistant neutrophilic airway inflammation[45, 46]. Furthermore, combined targeting of T_H_2 and T_H_17 inflammation has been suggested based on race in treatment of atopic dermatitis[13].

In summary, we have identified systemic cellular and cytokine/chemokine inflammatory profiles among inner-city African American children with Easy-to-Control and Difficult-to-Control asthma. Our study highlights the need to consider targeting multiple inflammatory pathways in the treatment of Difficult-to-Control pediatric asthma in the African American population.

## Supporting information

S1 Table Mean, lower limit of detection, and proportion of values above the lower limit of detection, overall and by inflammatory marker. (DOCX)

S2 Table Comparison of African American subjects in the full APIC study population and the cytokine subsample. (DOCX)

S1 Fig Replicate agreement by inflammatory marker. Scatter plot of replicate A versus replicate B. Each panel represents a different cytokine, and each circle represents a pair of replicate values. The forty-five-degree line through the origin represents perfect agreement. Annotated values are the concordance coefficient correlation and associated 95% confidence interval.
(TIF)

S2 Fig Cytokine distributions and proportion of cytokines above the lower limit of detection. Left and middle panels display the distribution of values on the original scale and a log-10 scale, respectively. The spread of values is represented by boxplots, where the black dot represents the median, the gray box represents the interquartile range, the whiskers extend to 1.5 times the interquartile range, and individual tick marks represent data points beyond the 1.5*IQR threshold. Right panel contains the percentage of values above the lower limit of detection.
(TIF)

S3 Fig Result of feature selection procedure. Each line represents one of the 38 inflammatory markers. A non-zero beta coefficient (y-axis) represents statistical significance. As lambda grows (x-axis moves to the right), the penalty on the coefficients increases, driving irrelevant predictors to zero. Lines are truncated at the optimal lambda selected by repeated cross-validation (-2.97). At this level, 12 significant cytokines remain. Line color represents association with Difficult-to-Control (red = negative, blue = positive).
(TIF)

S1 File Zip file of analysis data and metadata. The CSV file (anly_cyto_share.csv) contains the core data for the analysis population of N = 235. The structure of the data is 1 record per participant per cytokine. The corresponding PDF file (anly_cyto_share.pdf) contains metadata, including names, labels, and distributions of each variable.
(ZIP)
