# Supplementary material for: Endotypes of difficult-to-control asthma in inner-city African American children
Source: PLoS One. 2017 Jul 7;12(7):e0180778. doi: 10.1371/journal.pone.0180778 (PMC5501607; doi:10.1371/journal.pone.0180778)
Supplement: S1 Table — (DOCX) [file pone.0180778.s001.docx]

S1 Table: Mean, lower limit of detection, and proportion of values above the lower limit of detection, overall and by inflammatory mediator.

| Cytokine | Lower limit of detection (pg/ml) | Percent of values above the lower limit of detection | Geometric Mean (SD) |
| --- | --- | --- | --- |
| *All* | *--* | *77.5* | *--* |
| CXCL-1 | 9.9 | 100 | 2036.1 (1.6) |
| EGF | 2.8 | 100 | 99.6 (3.1) |
| EOTAXIN | 4.0 | 100 | 64.9 (1.7) |
| FGF2 | 7.6 | 100 | 59.6 (1.9) |
| FLT3L | 5.4 | 44.7 | 9.9 (3.6) |
| FRACTALKINE | 22.7 | 95.3 | 99 (2.7) |
| GCSF | 1.8 | 99.6 | 40.6 (2.3) |
| GM-CSF | 7.5 | 95.3 | 27.1 (2.1) |
| IFN-α2 | 2.9 | 96.6 | 45 (2.8) |
| IFN-γ | 0.8 | 94.9 | 13.7 (4.8) |
| IL-10 | 1.1 | 61.7 | 3 (4.4) |
| IL-12p40 | 7.4 | 56.2 | 15.6 (3.4) |
| IL12p70 | 0.6 | 83.4 | 3.4 (5.1) |
| IL-13 | 1.3 | 56.6 | 5.2 (6.5) |
| IL-15 | 1.2 | 43.0 | 1.8 (3) |
| IL-17A | 0.7 | 83.4 | 5.1 (6) |
| IL-1α | 9.4 | 48.9 | 19.5 (3.9) |
| IL-1β | 0.8 | 41.3 | 1.3 (3.3) |
| IL-1RA | 8.3 | 99.1 | 72.6 (2.7) |
| IL-2 | 1.0 | 54.0 | 2.3 (4) |
| IL-3 | 0.7 | 16.6 | 0.6 (1.5) |
| IL-4 | 4.5 | 23.8 | 6.1 (3.8) |
| IL-5 | 0.5 | 54.5 | 1 (3.4) |
| IL-6 | 0.9 | 51.5 | 3.1 (6.4) |
| IL-7 | 1.4 | 83.4 | 6.5 (3.2) |
| IL-8 | 0.4 | 99.1 | 20.3 (3.3) |
| IL-9 | 1.2 | 47.7 | 1.7 (2.6) |
| IP-10 | 8.6 | 100 | 307.8 (1.8) |
| MCP-1 | 1.9 | 100 | 393.7 (1.7) |
| MCP-3 | 3.8 | 94.9 | 19.9 (2.6) |
| MDC | 3.6 | 100 | 1936.9 (1.5) |
| MIP-1α | 2.9 | 95.3 | 18.8 (3.9) |
| MIP-1β | 3.0 | 100 | 50.9 (1.9) |
| SCD40L | 5.1 | 100 | 9149.9 (1.4) |
| TGF-α | 0.8 | 74.5 | 2.6 (3.1) |
| TNF-α | 0.7 | 98.3 | 11.2 (2.1) |
| TNF-β | 1.5 | 53.2 | 4.3 (5.5) |
| VEGF | 26.3 | 100 | 273.9 (2.3) |
