# Supplementary material for: Endotypes of difficult-to-control asthma in inner-city African American children
Source: PLoS One. 2017 Jul 7;12(7):e0180778. doi: 10.1371/journal.pone.0180778 (PMC5501607; doi:10.1371/journal.pone.0180778)
Supplement: S2 Table — (DOCX) [file pone.0180778.s002.docx]

S2 Table. Comparison of African American subjects in the full APIC study population and the cytokine subsample.^1,2^

|  | All APIC African Americans | | | | Cytokine Subsample | | | |
| --- | --- | --- | --- | --- | --- | --- | --- | --- |
|  | All  (N=311) | Difficult-to-Control  (N=173) | Easy-to-Control  (N=138) | P-value | All  (N=235) | Difficult-to- Control  (N=127) | Easy-to- Control  (N=108) | P-value |
| Sex - Male | 177 (56.9%) | 95 (54.9%) | 82 (59.4%) | 0.50 | 131 (55.7%) | 67 (52.8%) | 64 (59.3%) | 0.39 |
| Age at Screening (years) | 11.2 (3.06) | 10.9 (3.05) | 11.4 (3.06) | 0.14 | 11.0 (2.93) | 11.1 (2.96) | 11.0 (2.92) | 0.91 |
| BMI percentile at Screening^3^ | 88.0 [56.0;98.1] | 89.3 [61.9;98.5] | 81.2  [53.7;97.0] | 0.03 | 88.3 [60.3;98.2] | 89.0 [64.8;98.7] | 87.9 [56.5;97.2] | 0.13 |
| Income < $ 15,000 | 158 (51.0%) | 84 (48.8%) | 74 (53.6%) | 0.47 | 119 (50.9%) | 63 (50.0%) | 56 (51.9%) | 0.88 |
| Blood eosinophil count at enrollment (cells/mm^3^) | 317 (237) | 349 (258) | 276 (201) | 0.006 | 318 (236) | 351 (252) | 278 (210) | 0.02 |
| Blood neutrophil count at enrollment (cells/mm^3^) | 2966 (1678) | 3170 (1859) | 2707 (1381) | 0.01 | 3002 (1779) | 3248 (1998) | 2712 (1437) | 0.02 |

1. Unless otherwise noted, characteristics are compared using chi-square tests for categorical variables, and t-tests for continuous variables.
2. Unless otherwise noted, summary statistics are frequency (%) for categorical variables and mean and standard deviation for continuous variables.
3. Summarized using the median and inter-quartile range and tested using a Kruskal-Wallis test.
