# Supplementary figures and images for: Endotypes of difficult-to-control asthma in inner-city African American children
Source: PLoS One. 2017 Jul 7;12(7):e0180778. doi: 10.1371/journal.pone.0180778 (PMC5501607; doi:10.1371/journal.pone.0180778)

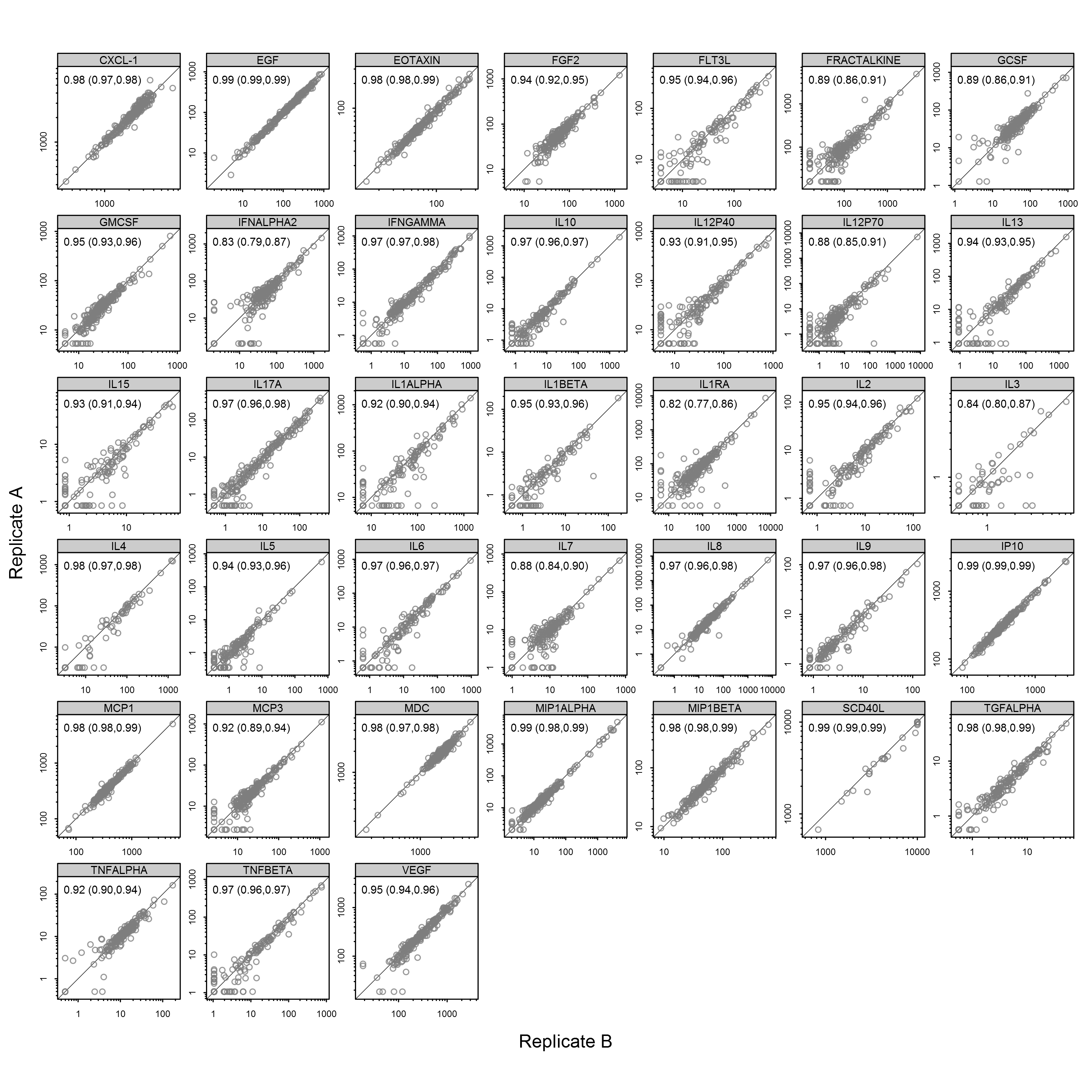

Supplement: S1 Fig — Scatter plot of replicate A versus replicate B. Each panel represents a different cytokine, and each circle represents a pair of replicate values. The forty-five-degree line through the origin represents perfect agreement. Annotated values are the concordance coefficient correlation and associated 95% confidence interval. (TIF) [file pone.0180778.s003.tif]

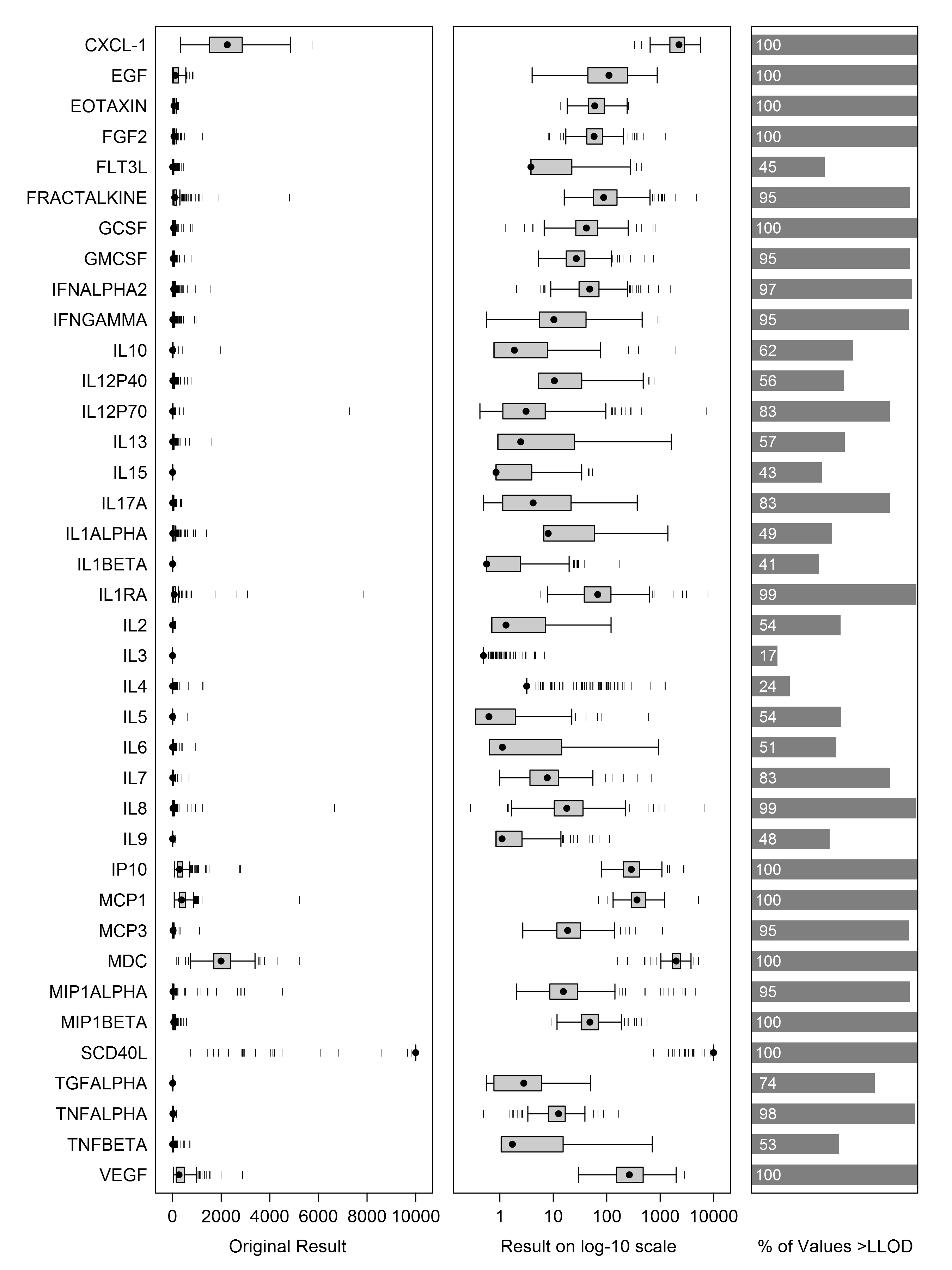

Supplement: S2 Fig — Left and middle panels display the distribution of values on the original scale and a log-10 scale, respectively. The spread of values is represented by boxplots, where the black dot represents the median, the gray box represents the interquartile range, the whiskers extend to 1.5 times the interquartile range, and individual tick marks represent data points beyond the 1.5*IQR threshold. Right panel contains the percentage of values above the lower limit of detection. (TIF) [file pone.0180778.s004.tif]

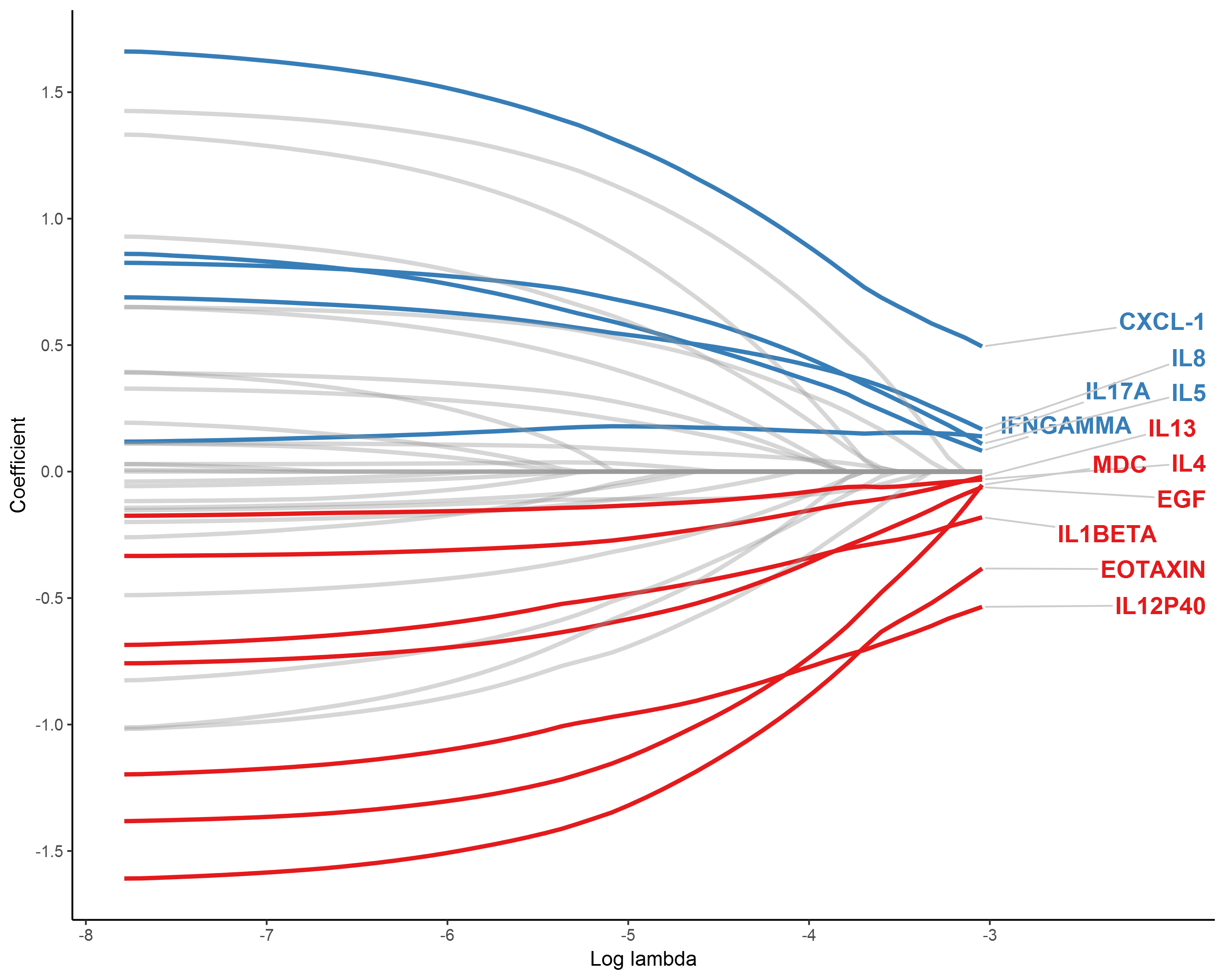

Supplement: S3 Fig — Each line represents one of the 38 inflammatory markers. A non-zero beta coefficient (y-axis) represents statistical significance. As lambda grows (x-axis moves to the right), the penalty on the coefficients increases, driving irrelevant predictors to zero. Lines are truncated at the optimal lambda selected by repeated cross-validation (-2.97). At this level, 12 significant cytokines remain. Line color represents association with Difficult-to-Control (red = negative, blue = positive). (TIF) [file pone.0180778.s005.tif]
