# Supplementary material for: Endotypes of difficult-to-control asthma in inner-city African American children
Source: PLoS One. 2017 Jul 7;12(7):e0180778. doi: 10.1371/journal.pone.0180778 (PMC5501607; doi:10.1371/journal.pone.0180778)
Supplement: S1 File — The CSV file (anly_cyto_share.csv) contains the core data for the analysis population of N = 235. The structure of the data is 1 record per participant per cytokine. The corresponding PDF file (anly_cyto_share.pdf) contains metadata, including names, labels, and distributions of each variable. (ZIP) [file pone.0180778.s006.zip › anly_cyto_share.pdf]

## 10 Variables 8930 Observations

### id : Subject ID

| n | missing | unique | sd | Mean | .05 | .10 | .25 | .50 | .75 | .90 | .95 |
|---|---|---|---|---|---|---|---|---|---|---|---|
| 8930 | 0 | 235 | 67.8 | 118 | 12 | 24 | 59 | 118 | 177 | 212 | 224 |

```
lowest :   1   2   3   4   5, highest: 231 232 233 234 235
```

### trt_class : Final Protocol Classification

| n | missing | unique |
|---|---|---|
| 8930 | 0 | 2 |

```
Difficult-to-control (4826, 54%), Easy-to-control (4104, 46%)
```

### age : Age of participant at screening visit

| n | missing | unique | sd | Mean | .05 | .10 | .25 | .50 | .75 | .90 | .95 |
|---|---|---|---|---|---|---|---|---|---|---|---|
| 8930 | 0 | 12 | 2.93 | 11 | 7 | 7 | 9 | 11 | 13 | 15 | 16 |

| | 6 | 7 | 8 | 9 | 10 | 11 | 12 | 13 | 14 | 15 | 16 | 17 |
|---|---|---|---|---|---|---|---|---|---|---|---|---|
| Frequency | 418 | 722 | 1064 | 760 | 874 | 1140 | 1330 | 646 | 570 | 722 | 380 | 304 |
| % | 5 | 8 | 12 | 9 | 10 | 13 | 15 | 7 | 6 | 8 | 4 | 3 |

### gender : Gender

| n | missing | unique |
|---|---|---|
| 8930 | 0 | 2 |

```
Female (3952, 44%), Male (4978, 56%)
```

### eosinophil_total : Total Eosinophils - per ul

| n | missing | unique | sd | Mean | .05 | .10 | .25 | .50 | .75 | .90 | .95 |
|---|---|---|---|---|---|---|---|---|---|---|---|
| 8930 | 0 | 75 | 235 | 318 | 70 | 100 | 150 | 250 | 407 | 600 | 800 |

```
lowest :   0.0    8.1   10.0   40.0   50.0
highest: 900.0 1000.0 1100.0 1180.0 1300.0
```

### neutrophil_total : Total Neutrophils - per ul

| n | missing | unique | sd | Mean | .05 | .10 | .25 | .50 | .75 | .90 | .95 |
|---|---|---|---|---|---|---|---|---|---|---|---|
| 8930 | 0 | 137 | 1776 | 3002 | 1100 | 1400 | 1840 | 2600 | 3580 | 5040 | 6600 |

```
lowest :  400   757   850  1000  1010
highest: 6980  7830  7900  8380 16900
```

### cytokine : Cytokine

| n | missing | unique |
|---|---|---|
| 8930 | 0 | 38 |

```
lowest : CXCL-1   EGF      EOTAXIN  FGF2     FLT3L
highest: SCD40L   TGFALPHA TNFALPHA TNFBETA  VEGF
```

### result : Cytokine result (pg/ml)

| n | missing | unique | sd | Mean | .05 | .10 | .25 | .50 |
|---|---|---|---|---|---|---|---|---|
| 8930 | 0 | 5967 | 1615 | 437 | 0.566 | 0.778 | 3.182 | 18.705 |

| .75 | .90 | .95 |
|---|---|---|
| 89.263 | 574.215 | 2240.913 |

```
lowest : 2.83e-01 3.54e-01 4.24e-01 4.32e-01 4.42e-01
highest: 7.86e+03 8.58e+03 9.66e+03 9.81e+03 1.00e+04
```

### result_l : Cytokine result (pg/ml) - log10

| n | missing | unique | sd | Mean | .05 | .10 | .25 | .50 | .75 | .90 | .95 |
|---|---|---|---|---|---|---|---|---|---|---|---|
| 8930 | 0 | 5967 | 1.08 | 1.31 | -0.247 | -0.109 | 0.503 | 1.272 | 1.951 | 2.759 | 3.350 |

```
lowest : -0.548 -0.452 -0.372 -0.365 -0.355
highest:  3.896  3.934  3.985  3.991  4.000
```

### result_detect: Cytokine result > LLOD?

| n | missing | unique | Sum | Mean |
|---|---|---|---|---|
| 8930 | 0 | 2 | 6925 | 0.775 |

Rho Giving flight to research
